# Natural Mediterranean Spotted Fever Foci, Qingdao, China

**DOI:** 10.3201/eid2812.221097

**Published:** 2022-12

**Authors:** Xiao-Lan Gu, Rui Wang, Chuan-Min Zhou, Jiang-Tao Cui, Ze-Min Li, Ze-Zheng Jiang, Bang Li, Qiu-Ming Peng, Wen-Kang Zhang, Hui-Ju Han, Xue-Jie Yu

**Affiliations:** Wuhan University, Wuhan, China (X.-L. Gu, R. Wang, C.-M. Zhou, Z.-M. Li, Z.-Z. Jiang, B. Li, Q.-M. Peng, W.-K. Zhang, H.-J. Han, X.-J. Yu);; Laixi City for Disease Control and Prevention, Qingdao, China (J.-T. Cui)

**Keywords:** Mediterranean spotted fever, Rickettsia conorii, bacteria, zoonoses, vector-borne infections, rodents, *Apodemus agrarius*, mouse, Indian tick typhus, China

## Abstract

We sequenced DNA from spleens of rodents captured in rural areas of Qingdao, East China, during 2013–2015. We found 1 *Apodemus agrarius* mouse infected with *Rickettsia conorii*, indicating a natural Mediterranean spotted fever foci exists in East China and that the range of *R. conorii* could be expanding.

Mediterranean spotted fever (MSF) is an acute febrile, zoonotic disease caused by the bacterium *Rickettsia conorii* that is transmitted to humans by the brown dog tick, *Rhipicephalus sanguineus* (*1*). MSF was described from the Mediterranean region in 1910 (*2*); a similar disease, known as Indian tick typhus (ITT), was described in 1925 (*3*). The causative agent of ITT was later confirmed to be *R. conorii* (*4*). Since 1990, the natural foci of MSF has continued to expand into the Middle East, Africa, and central Europe (*5*). *R. conorii* ITT has been detected in ticks in Xinjiang Province in West China (*6*), and an MSF case recently was reported in Shandong Province in East China (*7*).

The city of Qingdao, located in the southeast part of Shandong Province, is on the pacific coast of East China (Figure 1). Qingdao has a temperate monsoonal climate that is ideal for rodent propagation, and many natural-focal diseases caused by *Rickettsia* spp., *Orientia tsutsugamushi*, and severe fever with thrombocytopenia syndrome virus (*8*–*10*). We used PCR amplification to investigate whether rodents in the region are infected with *Rickettsia* spp. and unexpectedly discovered *R. conorii*.

**Figure 1:**
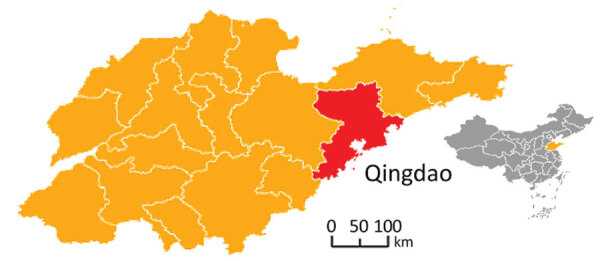
Location of rodent sampling sites in a study of natural Mediterranean spotted fever foci, Qingdao, Shandong Province, China. Inset map shows location of Shandong Province in China. Rodent species collected included striped field mice (*Apodemus agrarius*), Chinese hamsters (*Cricetulus barabensis*), house mice (*Mus musculus*), brown rats (*Rattus norvegicus*), greater long-tailed hamsters (*Cricetulus triton*), and Chinese white-bellied rats (*Niviventer confucianus*).

## The Study

We performed a retrospective study by testing rodents captured from Huangdao District, Qingdao, China, during July–October every year from 2013–2015. The rodent collections were previously described (*11*). We aseptically collected rodent spleens and stored at −80°C. Animal use and sample collection were approved by the ethics committee of Medical School, Shandong University (approval no. 20150501), and performed in accordance with Shandong University Guidelines on the Care and Use of Laboratory Animals.

We extracted DNA from homogenized rodent spleen tissues by using the QIAamp DNA Mini Kit (QIAGEN, https://www.qiagen.com). We initially performed nested PCR on all rodents with 17-kDa antigen (*htrA*), then we further tested the PCR-positive samples by using primers of 16S ribosomal RNA (*rrs*) and outer membrane protein A and B (*ompA* and *ompB*) genes (*12*,*13*) (Table 1). We used nuclease-free water as a negative control in each experiment. We performed DNA extraction, PCR amplification, and PCR product analysis in separate rooms to avoid false-positive results. We visualized PCR products in 1.0%–1.5% agarose gels based on the length of amplified DNA segments. We excised and extracted expected DNA bands by using a gel extraction kit (Tsingke Biotech, https://tsingke.com). We cloned purified PCR products into T-Vector pMD19 (TaKaRa Bio, Inc., https://www.takara-bio.com). Both strands were sequenced by Sangon Biotech (https://www.sangon.com).

**Table 1:** Primers used for amplification of spotted fever group *Rickettsia* from natural Mediterranean spotted fever foci, Qingdao City, China*

Target gene	Primer	Nucleotide sequence, 5′ → 3′	Length, bp	Reference
17 kDa antigen	F1	CATTGTCCGTCAGGTTGGCG	371	(*12*)
	R1	GGAACACTTCTTGGCGGTG		
	F2	AACCGTAATTGCCGTTATCCGG	214	
	R2	GCATTACTTGGTTCTCAATTCGG		
16S rRNA	F1	TGATCCTGGCTCAGAACGAAC	1,486	(*12*)
	R1	TAAGGAGGTAATCCAGCCGC		
	F2	AACACATGCAAGTCGRACGG	1,371	
	R2	GGCTGCCTCTTGCGTTAGCT		
Outer membrane protein A	F	ATGGCGAATATTTCTCCAAAA	536	(*13*)
	R	AGTGCAGCATTCGCTCCCCCT		
Outer membrane protein B	F1	ATATGCAGGTATCGGTACT	1,355	(*12*)
	R1	CCATATACCGTAAGCTACAT		
	F2	GCAGGTATCGGTACTATAAAC	843	
	R2	AATTTACGAAACGATTACTTCCGG		

*Samples studied were from rodents collected in Huangdao District, Qingdao, China, during July–October every year in 2013–2015. The rodent collection was described in a previous study (*11*). F, forward; R, reverse.

We edited DNA sequences by using DNAStar software (https://www.dnastar.com) to remove primers and analyzed sequences in BLAST (https://blast.ncbi.nlm.nih.gov/Blast.cgi) to compare with GenBank sequences. We constructed a phylogenetic tree by using the maximum-likelihood method with the Kimura 2-parameter model in MEGA version 7 (https://www.megasoftware.net), and we calculated bootstrap values with 1,000 replicates to determine the relative support for clades in the trees.

We used a total of 121 rodents in this study, including 60 striped field mice (*Apodemus agrarius*), 19 house mice (*Mus musculus*), 16 Chinese hamsters (*Cricetulus barabensis*), 10 brown rats (*Rattus norvegicus*), 8 greater long-tailed hamsters (*Cricetulus triton*), and 8 Chinese white-bellied rats (*Niviventer confucianus*) (Table 2). Among the rodents, 81.82% (99/121) were captured outdoors and 18.18% (22/121) indoors.

**Table 2:** Number and location of rodents collected in a study of natural Mediterranean spotted fever foci, Qingdao, China*

Species, no.	Outdoors	Indoors	Total
Striped field mice (*Apodemus agrarius*)	59	1	60
Chinese hamsters (*Cricetulus barabensis*)	16	0	16
House mice (*Mus musculus*)	8	11	19
Brown rats (*Rattus norvegicus*)	0	10	10
Greater long-tailed hamsters (*Cricetulus triton*)	8	0	8
Chinese white-bellied rats (*Niviventer confucianus*)	8	0	8
Total	99	22	121

*Rodents were collected in Huangdao District, Qingdao, China, during July–October every year in 2013–2015. The rodent collection was described in a previous study (*11*).

PCR amplification indicated that 1 *A. agrarius* mouse captured outdoors was positive for a *Rickettsia* species and the other 120 rodents were negative for *Rickettsia* by *htrA* primers. We further amplified the spleen tissue of the PCR-positive mouse by using *rrs*, *ompA*, and *ompB* primers. All 3 pairs of primers generated positive PCR products. Because the PCR fragment was too short and the sequence was conserved, we could not differentiate *Rickettsia* species by phylogenetic analysis of the *htrA* gene. However, the *rrs* gene sequence obtained in this study was 100% identical to *R. conorii* strains in GenBank (1,185/1,185 bp for accession no. KU364355, 1,267/1,267 bp for accession no. KY069267) that were obtained from *Rh. turanicus* ticks in Xinjiang, China. The obtained *rrs* sequence also matched 100% (1,331/1,331 bp) with a GenBank *R. conorii* strain from India (accession no. L36107), and an *R. conorii* Malish 7 strain (accession no. NR_041934). The *ompA* sequence from the mouse was 100% identical to *R. conorii* strains from *Rh. turanicus* ticks (494/494 bp for GenBank accession nos. MF002512 and KY069258) and an *R. conorii* strain from a human blood sample (449/449 bp for accession no. MG190328) from Xinjiang, China. The obtained *ompA* sequence was also 100% (494/494 bp) identical to GenBank *R. conorii* strains from India (accession no. U43794) and Italy (accession no. JN944636). The *ompB* amplified from the mouse was 99.87% (798/799 bp) homologous to the corresponding sequences of *R. conorii* from *Rh. turanicus* ticks from Xinjiang, China (GenBank accession nos. MF002514 and KY069249) and an *R. conorii* strain from India (GenBank accession no. AF123726).

**Figure 2:**
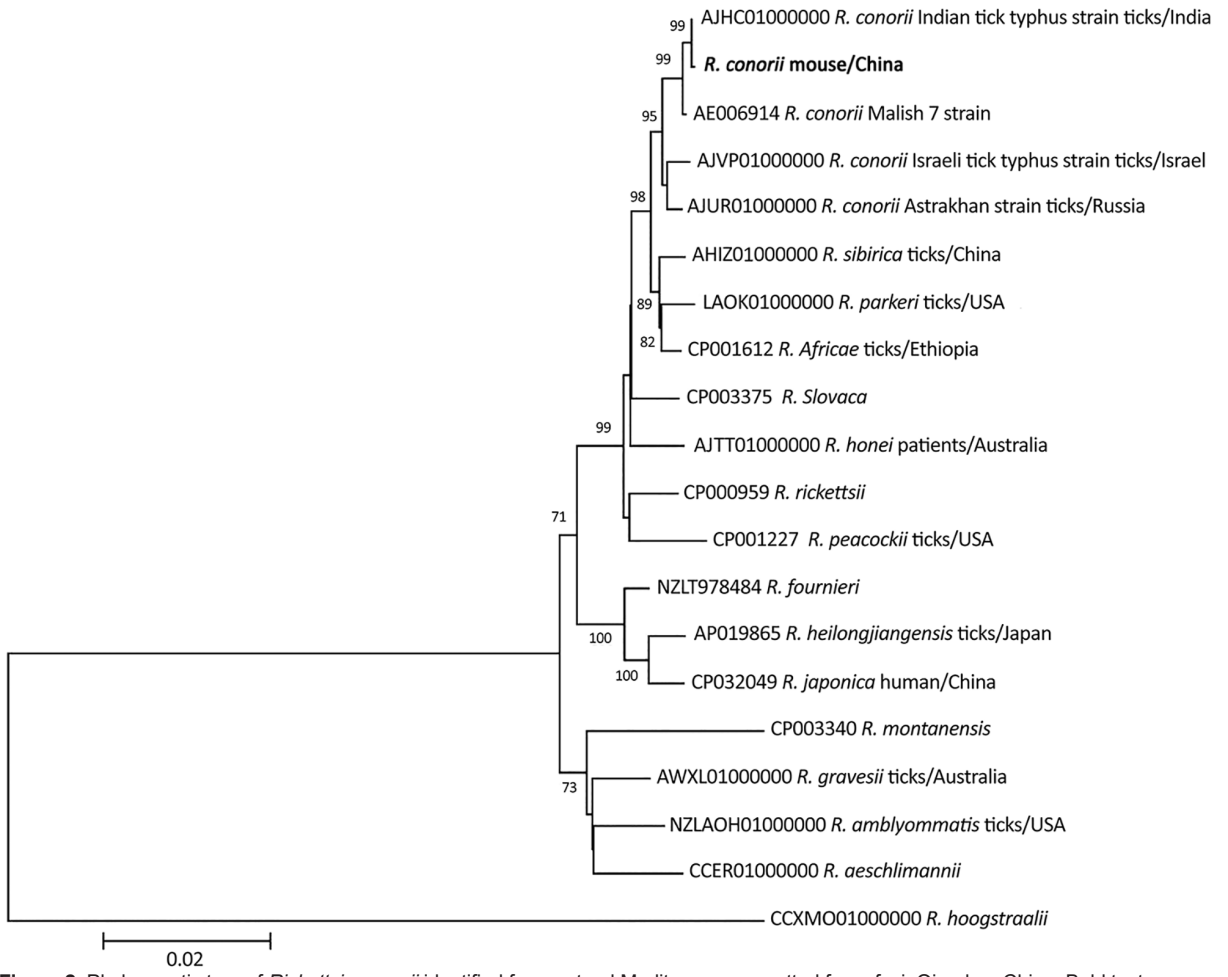
Phylogenetic tree of *Rickettsia conorii* identified from natural Mediterranean spotted fever foci, Qingdao, China. Bold text indicates *R. conorii* obtained from a striped field mouse (*Apodemus agrarius*) captured in 2015. The maximum-likelihood tree is based on the concatenated sequences of *rrs*, *htrA*, *ompA*, and *ompB* genes of *Rickettsia* species. Numbers at the nodes indicated the percentage of bootstrap proportions with 1,000 replicates; only bootstrap values >70% are shown. The reference sequences are indicated by the GenBank accession number, name of species, host, and country of isolation. Scale bar indicates nucleotide substitutions per site.

A phylogenetic tree of the concatenated sequences of the 4 genes, *rrs* (1,331 bp), *htrA* (169 bp), *ompA* (494 bp), and *ompB* (844 bp), showed that the rickettsial sequence from this study clustered with *R. conorii* ITT from ticks in India (GenBank accession no. AJHC01000000) and within the same clade as the *R. conorii* Malish 7 strain (GenBank accession no. AE006914), *R. conorii* Israel tick typhus strain (GenBank accession no. AJVP01000000), and *R. conorii* Astrakhan strain (GenBank accession no. AJUR01000000) (Figure 2). We deposited nucleotide sequence data from this study into GenBank (accession no. OM230141 for *rrs*, OM234678 for *htrA*, OM234679 for *ompA*, and OM234680 for *ompB* genes).

## Conclusions

We identified *Rickettsia* spp. in a striped field mouse captured in Qingdao in East China. Phylogenetic analysis showed that the *Rickettsia* species we detected was identical in multiple gene sequences to *R. conorii* ITT, indicating the strain we identified is *R. conorii*. Our results could not be caused by PCR contamination because we do not have an *R. conorii* strain nor its DNA in our laboratory.

The prevailing vector of *R. conorii* is the brown dog tick, *Rh. sanguineus* (*2*). The widespread distribution of *R. conorii* might be related to the worldwide spread of its vector tick among dogs (*14*). *R. conorii* ITT sequences have been reported in *Rh. turanicus* ticks from Xinjiang Province in West China (*6*). We identified *R. conorii* in 1 rodent in Qingdao, located in the eastern part of Shandong Province. Another recent study reported *R. conorii* genomic sequences in a patient in the western part of Shandong Province (*7*). These results demonstrate that the endemic area of *R. conorii* either recently expanded into Shandong Province in East China or *R. conorii* has existed in East China but was not detected before.

We speculate that the expansion of MSF foci in China is caused by transportation of dogs from West China to East China (*15*), contributing to the spread of brown dog ticks and, thus, *R. conorii*. The tick vector of *R. conorii* in Shandong Province has not been identified.

In conclusion, we confirmed *R. conorii* infection in 1 rodent from Qingdao in East China. Further studies are needed to determine the epidemiology of *R. conorii* in East China. Our study increases our knowledge about the distribution of *R. conorii*. Identification of MSF foci in East China could indicate that the range of *R. conorii* and its tick vector are expanding. 
